# Association of clozapine-related metabolic disturbances with CYP3A4 expression in patients with schizophrenia

**DOI:** 10.1038/s41598-020-78474-0

**Published:** 2020-12-04

**Authors:** Ádám Menus, Ádám Kiss, Katalin Tóth, Dávid Sirok, Máté Déri, Ferenc Fekete, Gábor Csukly, Katalin Monostory

**Affiliations:** 1 Department of Psychiatry and Psychotherapy, Semmelweis University, Balassa 6, 1082 Budapest, Hungary; 2 Institute of Enzymology, Research Centre for Natural Sciences, Magyar tudósok 2, 1117 Budapest, Hungary; 3 Toxi-Coop Toxicological Research Center, Magyar jakobinusok 4/B, 1122 Budapest, Hungary

**Keywords:** Medical research, Molecular medicine

## Abstract

Clozapine is effective in treatment-resistant schizophrenia; however, adverse effects often result in discontinuation of clozapine therapy. Many of the side-effects are associated with pharmacokinetic variations; therefore, the expression of major clozapine-metabolizing enzymes (CYP1A2, CYP3A4) in patients may predict development of adverse effects. In patients with schizophrenia (N = 96), development of clozapine concentration-dependent metabolic side-effects was found to be associated with pharmacokinetic variability related to CYP3A4 but not to CYP1A2 expression. In low CYP3A4 expressers, significant correlation was detected between fasting glucose level and clozapine concentration; moreover, the incidence of abnormal glucose level was associated with exaggerated clozapine concentrations (> 600 ng/ml). In low CYP3A4 expressers, exaggerated concentrations were more frequently observed than in normal/high expressers. Moderate/high risk obesity (BMI ≥ 35) more frequently occurred in low CYP3A4 expresser patients than in normal/high expressers. In patients with normal/high CYP3A4 expression and consequently with extensive clozapine-metabolizing capacity, norclozapine/clozapine ratio correlated with fasting glucose levels, triglyceride concentrations and BMI. Low CYP3A4 expression often resulting in exaggerated clozapine concentrations was considered to be as an important risk factor for some concentration-dependent adverse effects as normal/high CYP3A4 expression evoking high norclozapine/clozapine ratios. CYP3A4-status can identify patients with increased risk for metabolic side-effects and prevent their development by careful therapeutic strategy.

## Introduction

Clozapine, the first atypical antipsychotic, is indicated in patients with treatment-resistant schizophrenia and in those displaying severe and untreatable neurological adverse reactions to other antipsychotics^1–3^. According to CATIE study “for patients with schizophrenia who prospectively failed to improve with an atypical antipsychotic, clozapine was more effective than switching to another newer atypical antipsychotic”^4^. Clozapine has a low incidence of parkinsonism and other extrapyramidal side-effects, and does not increase the prolactin serum levels^5,6^. Despite its unique effectiveness, the clinical use of clozapine is limited because of its side-effects, especially because of the risk for agranulocytosis, seizure, myocarditis and cardiomyopathy^7^. Although the incidence of agranulocytosis is low (< 1%), this life-threatening side-effect is dose-independent. Therefore, regular haematological monitoring is mandatory during clozapine therapy^8^. Clozapine-associated myocarditis and cardiomyopathy are also rare; however, lethal outcome occurs in about 25% of cases^9^. The risk of epileptic seizures has been reported to increase with high daily doses associated with exaggerated serum concentrations^10^. The most commonly encountered side-effects, such as sedation, hypotension, increased heart rate, hypersalivation, constipation, weight gain and metabolic side-effects, are typically less serious; however, they often lead to discontinuation of clozapine by patients^11,12^. Among antipsychotics, clozapine has the highest propensity to induce rapid weight gain, hyperlipidaemia, hyperglycaemia and type 2 diabetes mellitus. Leadbetter et al.^13^ reported that during the first 16 weeks of clozapine therapy, 38% of patients showed marked (> 10% weight gain) and 29% moderate weight gain (5–10%). The excessive weight gain seems to be more frequent in female patients than in males^14^. The prevalence of metabolic syndrome is significantly higher in patients on clozapine therapy than in general population, and the risk considerably increases with age, body mass index (BMI) and duration of treatment^11,15^. Treatment duration was found to be a potent risk factor for hyperglycaemia and diabetes mellitus. Several studies have investigated the association between clozapine daily dose and metabolic side-effects; however, the results are controversial because clozapine plasma concentration rather than clozapine daily dose seems to have an impact on metabolic changes^16^.

Therapeutic drug monitoring of clozapine concentration is strongly recommended due to the significant inter-individual variability of plasma concentrations at the same daily dose^17^. The therapeutic range of clozapine steady-state concentration is 350–600 ng/ml; however, lower concentrations (200–300 ng/ml) were demonstrated to be as effective as higher levels (300–450 ng/ml)^18^, whereas adverse effects are more frequent at high concentrations^19,20^. Clozapine is metabolized in the liver by various cytochrome P450 enzymes (CYP1A2, CYP3A4, CYP2C19, CYP2D6) and flavin-containing monooxygenase 3^21,22^; however, CYP1A2 and CYP3A4 enzymes seem to be primarily responsible for the main metabolic pathways, forming two major metabolites, the pharmacologically active norclozapine and the inactive clozapine *N*-oxide^23,24^. In vitro evidence suggests that *N*-demethylation of clozapine to norclozapine is catalysed by both CYP1A2 and CYP3A4, whereas formation of clozapine *N*-oxide is primarily attributed to CYP3A4^23^. Although in vitro studies indicated the dominant role of CYP3A4 (30–45%) and minor contribution of CYP1A2 (10%) to clozapine clearance^22^, several clinical studies provided evidence that CYP1A2 was the major catalyst of clozapine metabolism^25–27^, while potential role of CYP3A4 in clozapine elimination was also emphasized^28^. Norclozapine has a pharmacodynamic profile somewhat different from that of clozapine, notably D_2_/D_3_ partial agonism, while clozapine displays D_2_/D_3_ inverse agonism^29^. Furthermore, both clozapine and norclozapine have antagonist effect on 5-HT_2C_ receptors, which likely contributes to clozapine-induced weight gain^11^.

Patients’ drug-metabolizing capacity is influenced by genetic factors and by several non-genetic factors, such as age, gender, co-medication, medical comorbidities, hormones (e.g. steroid hormones, insulin), smoking and nutrients (e.g. some components in broccoli, char-grilled meat, grapefruit or St. John’s wort). Genetic polymorphisms of *CYP1A2* and *CYP3A4* (CYP1A2*1C, CYP1A2*1F, CYP3A4*1B and CYP3A4*22) influence transcriptional expression and mRNA stability of CYP1A2 and CYP3A4; however, they seem to be of limited impact on drug metabolism^30^, and the substantial inter-individual variability in CYP1A2 and CYP3A activities cannot be explained exclusively by genetic polymorphisms. Non-genetic factors, such as transcriptional induction by CYP3A-inducers (e.g. carbamazepine or valproate) and by CYP1A2-inducing smoking strongly influence the expression levels and/or in vivo activities, and can mask the effects of genetic factors on CYP3A4 and CYP1A2 expression. Furthermore, gender-associated variations in clozapine levels were documented by several authors, being clozapine concentrations lower in male than in female patients^25,31,32^. The differences can be explained by hormonal balance, body composition, function of transporters and/or the activities of clozapine-metabolizing CYP1A2 and CYP3A4 enzymes^33,34^.

The clozapine-metabolizing capacity of the liver can be assessed by determining CYP genotypes and CYP expressions. We have previously described a complex diagnostic system (CYPtest) that identifies CYP polymorphisms and CYP mRNA expressions in leukocytes^35^. CYP1A2 and CYP3A4 mRNA levels in leukocytes have been demonstrated to inform about hepatic CYP1A2 and CYP3A4 activities. According to our previous study, CYP3A4 expression in patients with schizophrenia significantly correlated with clozapine plasma concentration, whereas no association was found between CYP1A2 expression and clozapine levels most probably because most of the patients expressed CYP1A2 at low level^28^. In the low CYP3A4 expresser group, the clozapine concentration was significantly higher, and the clozapine dose-requirement for therapeutic concentration was significantly lower compared to CYP3A4 normal and high expressers. Furthermore, the rate of both clozapine *N*-oxidation and *N*-demethylation pathways was significantly lower in patients expressing CYP3A4 at low level than in normal and high expressers.

Increasing awareness of prevention and treatment of metabolic syndrome associated with clozapine therapy is inevitable because the cardiovascular morbidity and mortality are higher in schizophrenia than in general populations^36^. The aim of the present study was to investigate the association of patients’ clozapine-metabolizing capacity with development of clozapine-related metabolic side-effects. Since several studies have previously supported the evidence that clozapine plasma concentration and norclozapine/clozapine ratio are related to metabolic side-effects, CYP genotypes and expression of the most relevant CYP enzymes involved in clozapine metabolism may provide information about the risk of metabolic disturbances. Determining the relevant CYP-status before the initiation of clozapine therapy may provide an appropriate tool for clinicians to reduce the risk of some of the side-effects by applying lower doses or using alternative antipsychotic treatment strategy in the susceptible group of patients. The complex associations of metabolic parameters (serum glucose, cholesterol and triglyceride levels), BMI, plasma concentrations of clozapine and its *N*-demethylated metabolite with CYP1A2 and CYP3A4 expressions were evaluated in the present study. CYP-status guided medication can be a step forward in personalized drug therapy that can contribute to a lower incidence of adverse effects and thus to a better quality of life for patients with schizophrenia.

## Materials and methods

### Patients and clozapine therapy

Ninety-six inpatients with schizophrenia (40%/60% male/female ratio) at the Department of Psychiatry and Psychotherapy, Semmelweis University (Budapest, Hungary) were enrolled in the study. Inclusion criteria were the clinical diagnosis of schizophrenia, an age of 18 years or older and stable clozapine therapy for more than one month. Exclusion criteria were major neurocognitive disorder, drug or alcohol addiction and substance use in the time of assessments or 6 months before enrolment. Other comorbidities were allowed. Altogether nine patients were on antidiabetic therapy and four patients took medication for hypercholesterinaemia. The potential role of patients’ CYP3A-status in clozapine pharmacokinetics in the group of patients was published earlier^28^. The present study was approved by the Hungarian Committee of Science and Research Ethics, Medical Research Council. The study was performed in accordance with the relevant guidelines and regulations (Act CLIV of 1997 on Health, decree 23/2002 of the Minister of Health of Hungary and the declaration of Helsinki). The patients met the criteria for schizophrenia based on the Structured Clinical Interview for Diagnostic and Statistical Manual of Mental Disorders^37^. Psychiatric symptoms based on the PANSS (Positive and Negative Syndrome Scale)^38^ were evaluated by trained psychiatrists. Patients’ demographic and clinical data (age, gender, bodyweight, smoking habit) as well as details of clozapine therapy were recorded (Table 1). Some of the patients received haloperidol, aripiprazole, risperidone or amisulpride in combination with clozapine. To ensure compliance, patients took their medication under supervision of a nurse.

**Table 1:** Patients’ demographic and clinical characteristics.

Parameter		N
Patients		96
Sex, male/female		38/58
Age (year)^a^		39 (18, 74)
Bodyweight (kg)^a^		75.6 (51, 128)
Body Mass Index (kg/m^2^)^a^		26.03 (17.0, 44.6)
Current smokers (n = 94)^b^		36
**PANSS**^**c**^		85.2 ± 23.5
	P1-7^c^	19.1 ± 6.6
	N1-7^c^	24.1 ± 8.4
	G1-16^c^	42.0 ± 12.1
Years from first diagnosis^a^		13.5 (0.08, 47)
Clozapine treatment duration (year)^a^		1 (0.08, 41)
Clozapine daily dose (mg)^c^		194.3 ± 130.5
**Serum levels (ng/ml)**^**c**^		
	Clozapine	268.5 ± 274.7
	Norclozapine	177.7 ± 161.7
Norclozapine/Clozapine ratio^c^		0.872 ± 0.582
Diabetes prior clozapine therapy		9
Fasting glucose level (mM, n = 83)^a, b^		4.7 (3.8, 9.2)
Total cholesterol level (mM, n = 78)^a, b^		4.5 (2.8, 7.1)
LDL cholesterol level (mM, n = 67)^a, b^		2.6 (1.7, 6.1)
HDL cholesterol level (mM, n = 69)^a, b^		1.4 (0.7, 2.3)
Triglyceride level (mM, n = 76)^a, b^		1.1 (0.4, 5.5)

^a^Median (min, max), ^b^missing or excluded data, ^c^mean ± SD.

### Reporting adverse events

Anticholinergic and anti-alpha-adrenergic adverse events were evaluated by the participant patients with the help of a structured self-assessment questionnaire edited by the study team. Patients were asked about the most common side-effects, such as blurred vision, palpitation, fatigue and dizziness, excessive salivation, dry mouth, constipation, urine retention and disturbances of memory and concentration (prospective data collection). The questionnaire consisted of yes/no questions (e.g. ‘Have you experienced the following symptom during the last week?’). For BMI, bodyweight and height were measured at the university clinic, and obesity was categorized into two categories according to severity, such as low risk obesity (35 > BMI ≥ 30 / class I obesity) and moderate/high risk obesity (BMI ≥ 35 / class II and III obesity) (39). Metabolic parameters like fasting glucose concentrations, serum triglyceride levels, total cholesterol, high density (HDL) and low density lipoprotein (LDL) cholesterol levels were measured in the central laboratory of Semmelweis University.

### Assaying CYP-status

Patients’ CYP-status was determined by *CYP1A2, CYP3A4* and *CYP3A5* genotyping and by analysing CYP1A2 and CYP3A4 expression in leukocytes. Genomic DNA and leukocytes were isolated from the peripheral blood samples as previously described by Temesvári et al.^35^. Hydrolysis single-nucleotide polymorphism analysis for CYP1A2*1C, CYP1A2*1F, CYP3A4*1B, CYP3A4*22 and CYP3A5*3 was performed using TaqMan probes (BioSearch Technologies, Novato, CA). For assaying CYP1A2 and CYP3A4 expressions, RNA was isolated from leukocytes, reverse transcribed into single-stranded cDNA using the Maxima First Strand cDNA Synthesis Kit (ThermoFisher Scientific, Waltham, MA), and then real-time PCR was performed using KAPA Fast Probes Mastermix (KAPA Biosystems, Cape Town, South Africa) and TaqMan probes. The quantities of CYP1A2 or CYP3A4 mRNAs relative to that of the housekeeping gene glyceraldehyde 3-phosphate dehydrogenase (GAPDH) were determined. GAPDH expression is constant in all cells and independent of experimental conditions; therefore, its expression was set to 1, and CYP mRNA levels were normalized by GAPDH expression. Three categories of CYP expressions were applied to describe low, normal and high expressers. The cut-off values for CYP mRNA levels in leukocytes have been previously established on the basis of the cut-off values for the hepatic CYP enzyme activities (CYP1A2 selective phenacetin *O*-dealkylation; CYP3A4 selective nifedipine oxidation or midazolam 1′- and 4-hydroxylation) in liver tissue donors. The cut-off values for CYP1A2 (10^−5^ and 5*10^−4^) and CYP3A4 (10^−6^ and 10^−4^) allowed a distinction between low, normal and high expressers^35^.

### Serum concentrations of clozapine and norclozapine

The blood samples were taken 12 h after the evening dose of clozapine. The steady-state concentrations of clozapine and norclozapine were determined by liquid chromatography-mass spectrometry. Chromatographic separation was performed using an Inertsil ODS-4 (75 × 2.1 mm, 3 µm) column (GL Sciences Inc., Tokyo, Japan) and mobile phases of acetonitrile and 0.1% formic acid in gradient running mode. The samples were analysed using positive electrospray ionization and multiple reaction monitoring mode for quantitation of the parent compound and its metabolite (*m/z* 327/270 and 327/192 for clozapine; *m/z* 313/270 and 313/192 for norclozapine)^28^.

### Statistical analysis

The effects of CYP1A2 and CYP3A4 expression on clozapine plasma concentrations [dichotomized as exaggerated (> 600 ng/ml) *vs* therapeutic (≤ 600 ng/ml) clozapine concentrations] were investigated by a logistic regression model (PROC LOGISTIC, Wald Chi-Square test). The association of CYP expression with norclozapine/clozapine ratios was analysed with Kruskal–Wallis non-parametric test, because the distribution of norclozapine/clozapine ratios deviated strongly from normal distribution. The effects of CYP1A2 and CYP3A4 expressions (low or normal/high expression) and clozapine dose on patient reported adverse effects, glucose levels [dichotomized as abnormal (≥ 5.8 mM) *vs* normal (< 5.8 mM) glucose levels] and obesity (yes/no) were also investigated by a logistic regression model (PROC LOGISTIC, Wald Chi-Square test). Effect sizes for CYP1A2 and CYP3A4 expression (low *vs* normal/high expression) in the logistic regression analyses are presented in terms of Odds Ratios (OR).

The relationship of serum glucose concentration, triglyceride level, LDL, HDL, and total cholesterol levels with clozapine dose and serum concentration was investigated by Pearson correlation (PROC CORR). The correlation of norclozapine/clozapine ratios with the same metabolic measures was investigated by Spearman correlation, because the distribution of norclozapine/clozapine ratios strongly deviated from normal distribution. These correlation analyses were performed separately in patients expressing CYP1A2 or CYP3A4 at normal/high and low level. Although 96 patients were involved in the study, some analyses evaluated data of patients less than 96, because some clinical data were missing due to the loss of follow up (e.g. continued treatment in other hospitals) or data of some patients were excluded due to diabetes mellitus existing prior clozapine therapy.

### Informed consent

Written informed consent was obtained from all participants.

## Results

**Figure 1:**
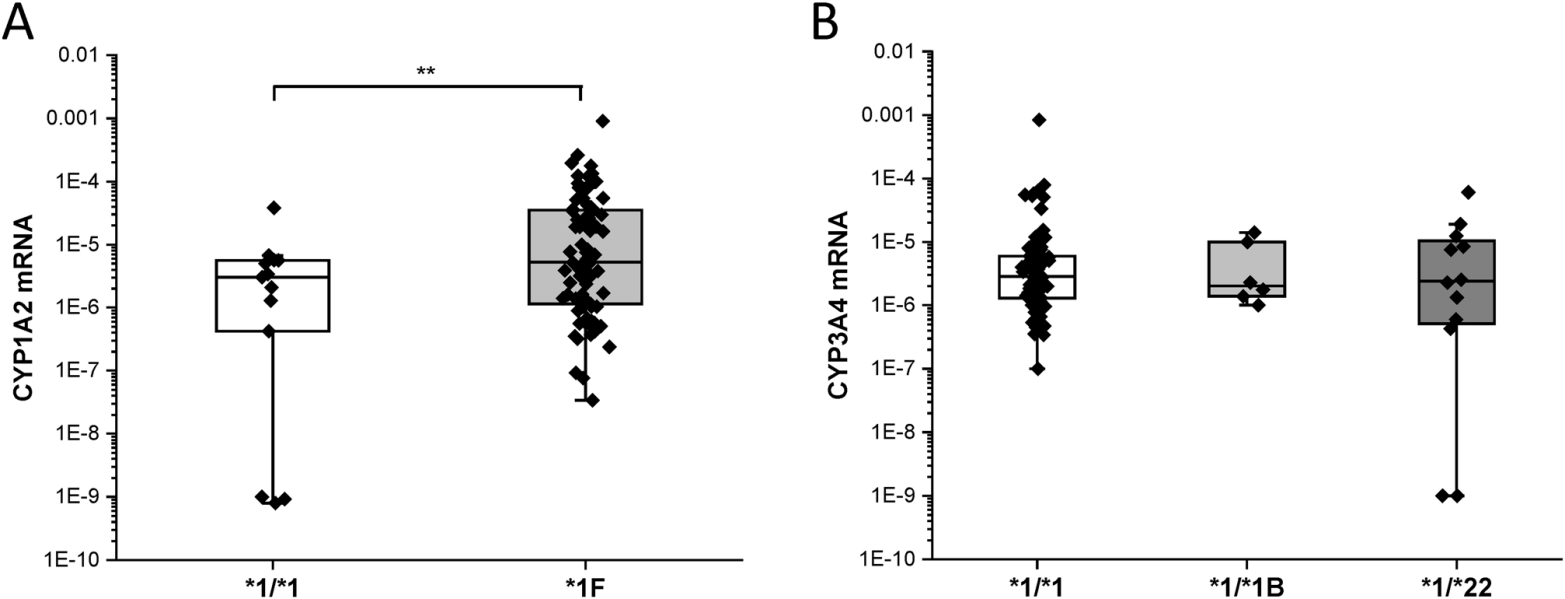
CYP1A2 and CYP3A4 expression in patients’ peripheral leukocytes (N = 96). (**A**) CYP1A2 mRNA levels in the patients carrying only wild-type *CYP1A2* allele (CYP1A2*1/*1) and in those carrying one or two CYP1A2*1F alleles. (**B**) CYP3A4 mRNA levels in the patients with CYP3A4*1/*1, CYP3A4*1/*1B and CYP3A4*1/*22 genotypes. **P = 0.001.

Of 96 patients, 82 carried one or two CYP1A2*1F alleles (CYP1A2*1/*1F, CYP1A2*1F/*1F, CYP1A2*1C/*1F) (Table 2), displaying allele frequency (66.1%) similar to that in Caucasian white populations (50–80%)^30^. CYP1A2 expression in patients’ leukocytes was found to be significantly associated with the presence of CYP1A2*1F allele (Fig. 1A), whereas CYP1A2*1C allele was identified only in one patient. CYP3A4*22 or CYP3A4*1B alleles, resulting in reduced and increased expression of CYP3A4, respectively, were identified in 18 patients; however, these alleles did not explain the inter-individual differences in CYP3A4 mRNA levels probably due to non-genetic factors (Fig. 1B). The hepatic CYP1A2 and CYP3A4 activities were therefore estimated from mRNA levels in patients’ leukocytes, categorizing the patients into low, normal and high expresser groups (Table 2).

**Table 2:** Patients’ CYP1A2 and CYP3A status.

CYP genotype	N	%
***CYP1A2***		
*1/*1	14	14.6
*1/*1F	36	37.5
*1F/*1F	45	46.9
*1C/*1F	1	1.0
***CYP3A4***		
*1/*1	78	81.3
*1/*1B	6	6.2
*1/*22	12	12.5
***CYP3A5***		
*1/*3	10	10.4
*3/*3	86	89.6
**CYP expression**		
**CYP1A2**		
Low expresser	62	64.6
Normal expresser	33	34.4
High expresser	1	1.0
**CYP3A4**		
Low expresser	23	24.0
Normal expresser	72	75.0
High expresser	1	1.0

### Association of CYP expression with clozapine and norclozapine concentrations

**Figure 2:**
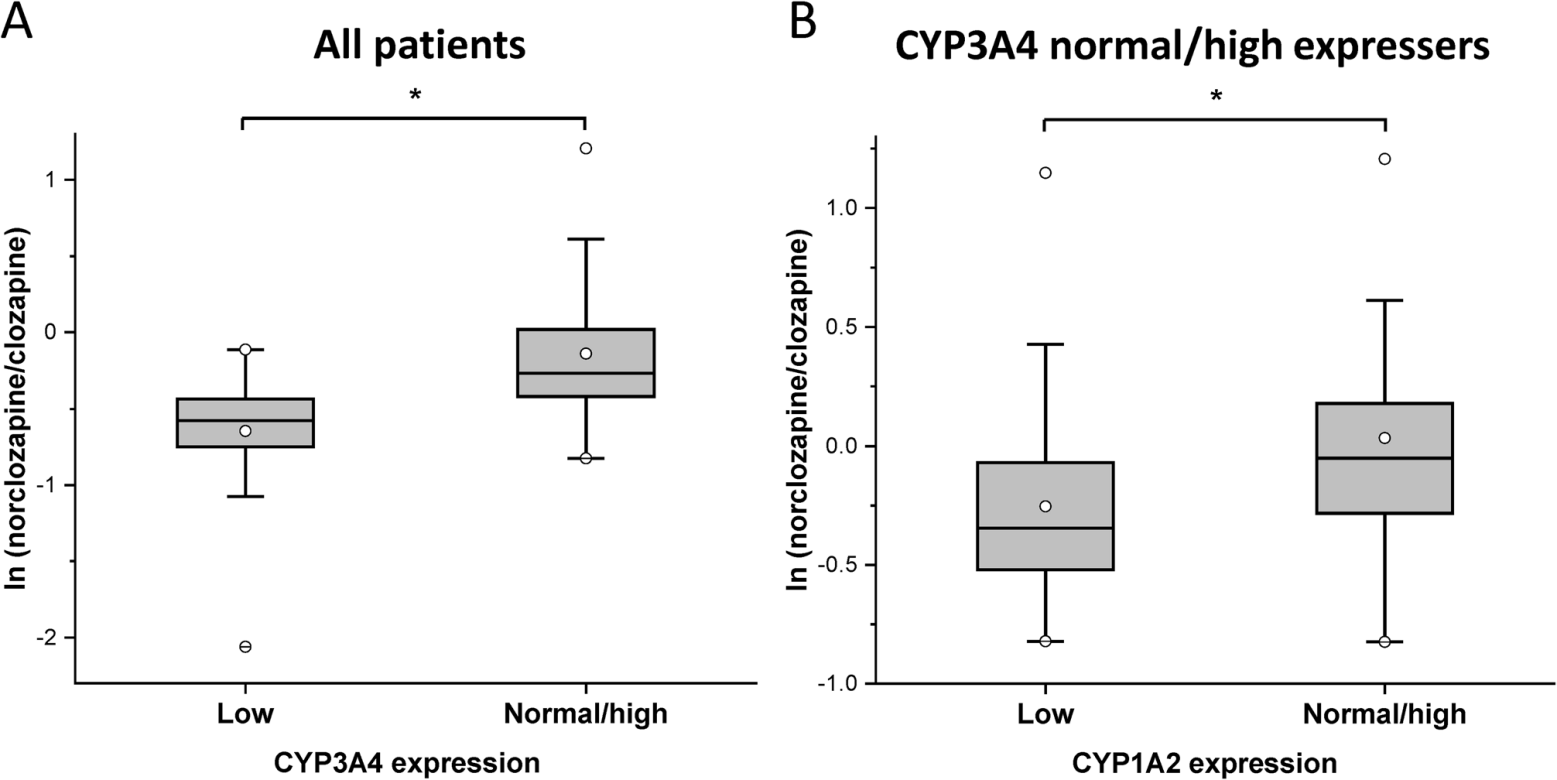
The influence of the patients’ CYP3A4 and CYP1A2 expressions on norclozapine/clozapine ratio. (**A**) Norclozapine/clozapine ratios in the patients with low and normal/high CYP3A4 expression (N = 96), and (**B**) norclozapine/clozapine ratios in the patients with normal/high CYP3A4 expression (N = 73) and with various CYP1A2 expression. * P < 0.001.

Exaggerated clozapine concentrations (> 600 ng/ml) were more frequently observed in patients with low CYP3A4 expression (22%) than in normal/high expressers (2.7%), indicating higher risk of clozapine concentrations over the therapeutic range for low CYP3A4 expressers [low *vs* normal/high expressers: OR = 9.8 (95%CL = 1.8–55.0), Wald ChiSq = 6.8, N = 96, P = 0.009]. Furthermore, strong association was observed between norclozapine formation and CYP3A4 expression [0.56 ± 0.17 *vs* 0.98 ± 0.62, Kruskal–Wallis ChiSq = 22.9, N = 96, P < 0.0001] (Fig. 2A), and further contribution of CYP1A2 to norclozapine production was also demonstrated [0.86 ± 0.55 *vs* 1.17 ± 0.70, Kruskal–Wallis ChiSq = 11.5, N = 73, P = 0.0007] (Fig. 2B).

### Association of metabolic parameters with CYP expression

No association was found between CYP1A2 or CYP3A4 expression and some metabolic parameters including blood glucose, triglyceride or cholesterol (total, HDL and LDL cholesterol) levels in patients (P > 0.1). BMI was evaluated only in those patients who took clozapine medication for at least one year (N = 87) in order to detect long-term effect of clozapine therapy on bodyweight. Obesity was categorized into two categories according to severity, such as low risk obesity (35 > BMI ≥ 30, class I obesity) and moderate/high risk obesity (BMI ≥ 35, class II and III obesity)^39^. The effect of CYP1A2 and CYP3A4 expressions on development of obesity was investigated in two separate logistic regression models (for the two levels of obesity) with clozapine dose and treatment duration as covariates. CYP1A2 expression appeared to be in no association with obesity (P > 0.1); furthermore, the frequency of patients with obesity was similar in the groups of patients expressing CYP3A4 at normal/high and low levels (18.5% of CYP3A4 normal/high expressers, 18.2% of CYP3A4 low expressers, Wald ChiSq = 0.1, N = 87, P = 0.98). However, moderate/high risk obesity was significantly more frequent in low CYP3A4 expressers than in normal expressers (13.6% of CYP3A4 low expressers, 1.5% of CYP3A4 normal/high expressers, OR = 13.5 [95%CL = 1.2–147.9], Wald ChiSq = 4.1, N = 87, P = 0.045). Clozapine dose and treatment duration longer than one year did not affect the frequency of obesity in any models (P > 0.3).

None of the adverse events reported by patients (N = 65) was influenced by the patients’ CYP1A2 expression (P > 0.1), and CYP3A4 expression had a marginally significant effect on constipation [47.1% in normal/high CYP3A4 expressers, 71.4% in low CYP3A4 expressers, OR = 3.6 (95%CL = 0.9–14.1), Wald ChiSq = 3.4, N = 65, P = 0.06] (Table 3), indicating that patients expressing CYP3A4 at low level tended to report constipation more frequently.

**Table 3:** Adverse effects reported by the patients with CYP3A4 normal/high and low expression.

Adverse event	Normal/high CYP3A4 expression (n = 51) [%]	Low CYP3A4 expression (n = 14) [%]	*P* value^a^
Thinking and concentration	66.7	71.4	0.76
Hypersalivation	64.7	71.4	0.52
Blurred vision	33.3	42.9	0.48
Constipation	47.1	71.4	**0.06**
Fatigue and dizziness	74.5	50.0	0.11

^a^*P* values are from a logistic regression model with CYP3A4 expression as predictor variable and occurrence of patient reported adverse effects as predicted variable.

*P* value indicating marginally significant effect is in bold.

### Association of metabolic parameters with clozapine therapy

**Figure 3:**
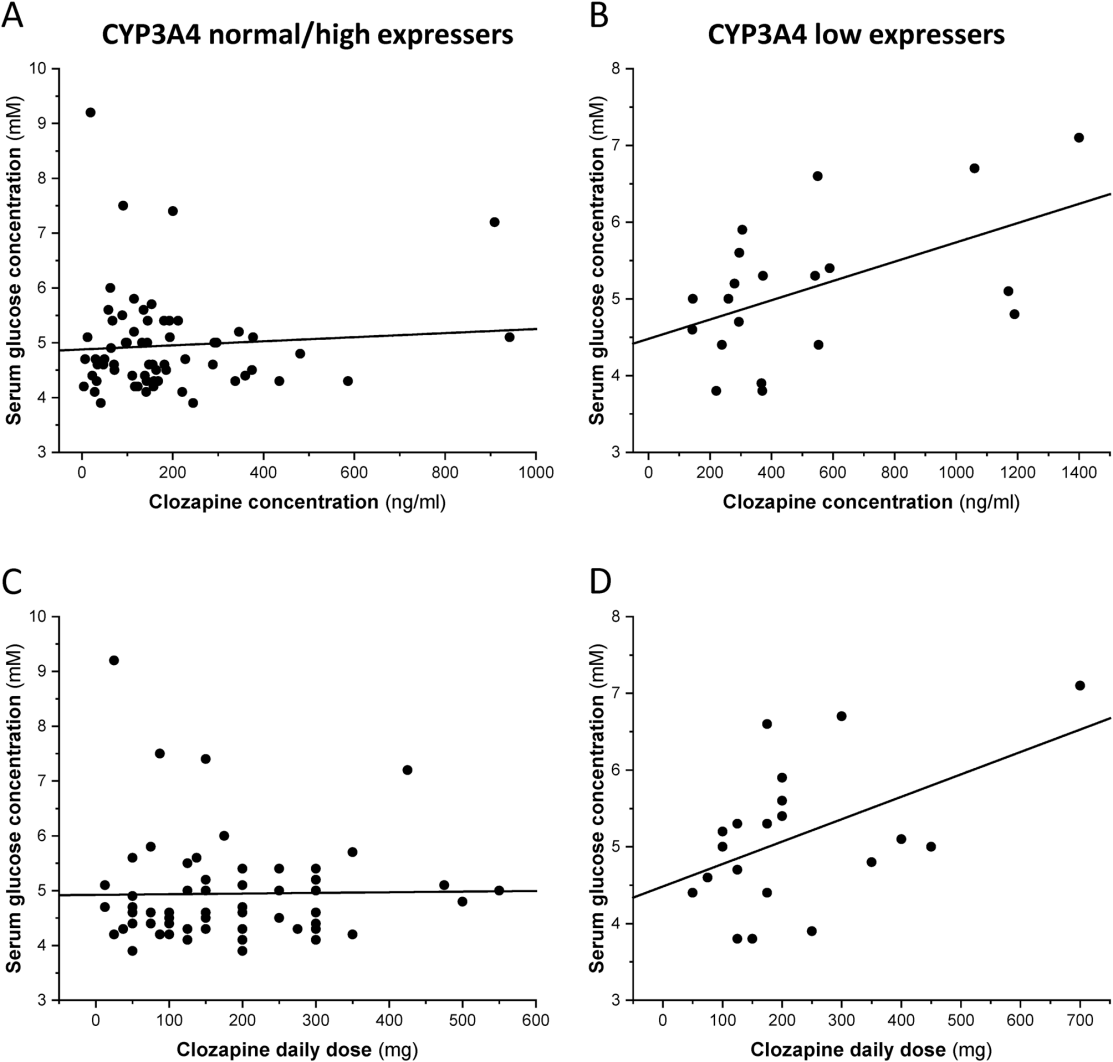
Association between serum glucose levels and clozapine steady-state concentrations (**A**,**B**) or clozapine daily doses (**C**,**D**) in CYP3A4 normal/high and low expressers.

**Figure 4:**
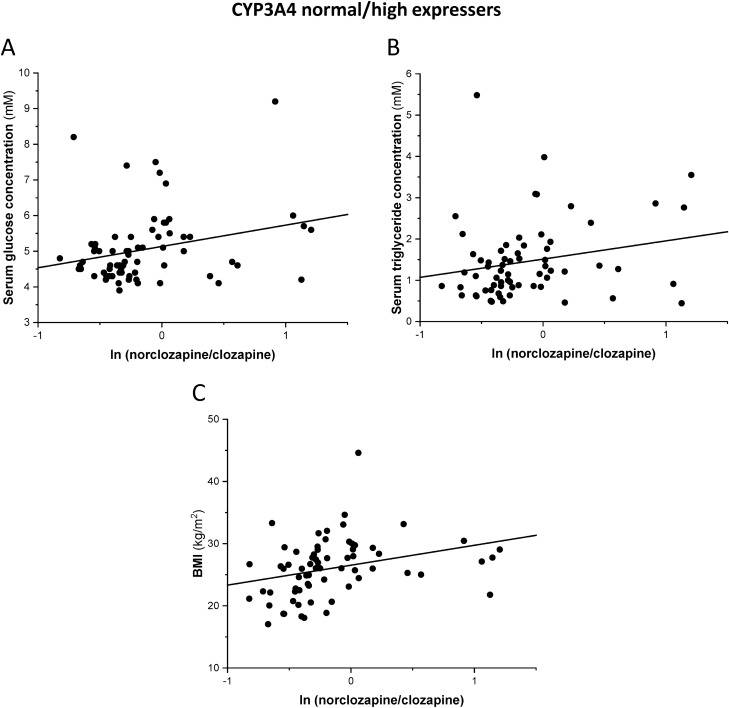
Correlation of serum glucose (**A**), triglyceride levels (**B**) and BMI (body mass index) (**C**) with norclozapine/clozapine ratios in CYP3A4 normal/high expressers treated with clozapine for at least one year.

Significant relationship of CYP3A4 expression and clozapine concentration with some metabolic parameters was observed (P < 0.05). In low CYP3A4 expressers, a significant correlation was found between clozapine serum concentration and blood glucose level (r = 0.52, N = 20, P = 0.02, missing or excluded: 3), while no correlation was observed in the patients expressing CYP3A4 at normal/high level (r = 0.07, N = 63, P = 0.58, missing or excluded: 10) (Fig. 3A,B). Similar correlation profile was demonstrated between the fasting glucose level and clozapine daily dose. In low CYP3A4 expresser patients, significant correlation was detected between glucose concentrations and the daily dose of clozapine (r = 0.49, N = 20, P = 0.03), while in those with normal/high CYP3A4 mRNA levels, no association was found (r = 0.02, N = 63, P = 0.9) (Fig. 3C,D). Patients with an existing diagnosis of diabetes mellitus (~ 9% in both groups, altogether 9 patients) were excluded from these analyses (Table 1). The incidence of abnormal fasting glucose levels (> 5.8 mM) was significantly higher in patients with exaggerated (> 600 ng/ml) clozapine serum concentrations compared with those displaying therapeutic clozapine concentrations [exaggerated clozapine concentrations: 50%, therapeutic clozapine concentrations: 8%, OR = 11.8 (95%CL = 1.9–71.9), Wald ChiSq = 7.2, N = 83, P = 0.007]. In normal/high CYP3A4 expressers, both fasting glucose levels (r = 0.27, N = 66, P = 0.03, missing or excluded: 10) and triglyceride levels (Spearman r = 0.26, N = 59, P = 0.048, missing or excluded: 14) significantly correlated with norclozapine/clozapine ratios (Fig. 4A,B), while no correlation was observed in low CYP3A4 expressers.

Blood concentrations of cholesterol (total, LDL, and HDL cholesterol) did not correlate with clozapine dose, clozapine serum concentration, or norclozapine/clozapine ratios (P > 0.1). Furthermore, serum triglyceride and cholesterol levels did not differ in the patients with exaggerated and therapeutic clozapine concentrations (P > 0.1). On the other hand, the norclozapine/clozapine ratio showed a strong correlation with BMI in the patients treated with clozapine for at least one year (Spearman r = 0.31, N = 87, P = 0.004). While this correlation was more pronounced in the normal/high CYP3A4 expresser group (Spearman r = 0.46, N = 65, P = 0.0001, Fig. 4C), a similar association was not observed in the low CYP3A4 expresser group (Spearman r = 0.08, N = 22, P = 0.72). Clozapine blood concentration did not correlate with BMI (P > 0.1).

Clozapine serum concentration had a significant effect on obstipation (Spearman r = 0.33, n = 65, P = 0.008) as reported by the patients. No further associations were found between clozapine concentration or norclozapine/clozapine ratio with patient reported adverse effects (P > 0.1).

### Discussion

Adverse events in patients during clozapine therapy have been reported to be associated with serum concentration rather than the dose of clozapine^20,40^. The steady-state clozapine concentrations have been demonstrated to be influenced by several CYP enzymes. A primary role in clozapine pharmacokinetics has been assigned to CYP1A2^26,41,42^; however, CYP3A4 has been emphasized to become an important determinant particularly in patients expressing CYP1A2 at low level^24,28,43^. According to the in vitro studies by Zhang et al.^24^, the relative activity of CYP1A2 and CYP3A4 is assumed to determine which enzyme has a greater role in clozapine metabolism. Ghassabian et al.^44^ showed significant correlation between the norclozapine/clozapine ratio and the activities of CYP1A2 and CYP3A4 using caffeine and midazolam as probe substrates, respectively. In smokers, the CYP1A2 activity is expected to be induced by the components of cigarette smoke^45^ and may potentially overshadow the importance of the CYP3A4 pathway.

Several studies found correlation between plasma concentrations of clozapine or norclozapine and metabolic abnormalities. Recently Lu et al.^46^ have reported that fasting glucose and triglyceride levels as well as weight gain were associated with high norclozapine levels, whereas no association was found with clozapine concentrations. Their results were partly confirmed by our findings regarding the association of norclozapine/clozapine ratios with fasting glucose and triglyceride levels or with BMI. However, the association was observed only in the patients expressing CYP3A4 at normal/high level, indicating that increased norclozapine production might have provoked the imbalance in glucose and lipid homeostasis. The facts that 5-HT_2C_ antagonism has been reported to be a mechanism underlying atypical antipsychotic-induced weight gain^47,48^, and that norclozapine has a greater antagonist effect on 5-HT_2C_ receptors than the parent compound^49^, could explain our findings of the positive correlation between BMI and norclozapine/clozapine ratios. The patients with high relative norclozapine concentrations (normal/high CYP3A4 expressers) appeared to tend to have substantial overweight as well as elevated fasting glucose and triglyceride levels. Co-administration of clozapine and fluvoxamine, the potent inhibitor of CYP1A2 and CYP3A4^50^, has been reported to result in reduction of norclozapine concentrations and as a consequence a decrease in the incidence of weight gain, hyperglycaemia, hypertriglyceridaemia, supporting norclozapine contribution to the development of metabolic side-effects^46^. On the other hand, clozapine concentration was found to be positively correlated with elevated insulin levels and higher frequency of insulin resistant state^51^. Similarly to these results, we found significant correlation between fasting blood glucose levels and clozapine serum concentrations in low CYP3A4 expresser patients. Furthermore, moderate/high risk obesity (BMI > 35) frequently occurred in patients expressing CYP3A4 at low level. Since low CYP3A4 expression often resulted in clozapine concentrations over therapeutic range (> 600 ng/ml), exaggerated clozapine levels can be considered to be as important risk factor for adverse effects as high norclozapine concentrations.

Besides the serotonergic side-effects, such as weight gain and glucose intolerance, many patients on clozapine therapy also reported cholinergic adverse effects^12^. In line with these previous studies, patients in the present investigation frequently reported cholinergic side-effects. More specifically some of these cholinergic adverse effects, such as constipation, were more frequent in patients presenting low CYP3A4 expression than in normal/high CYP3A4 expressers. Decreased gastrointestinal peristalsis and constipation as an anticholinergic adverse effect often occurs as a consequence of clozapine treatment. de Leon et al.^52^ reported that serum antimuscarinic activity, which correlated with clozapine plasma concentration, also showed a strong association with constipation.

Routine therapeutic drug monitoring generally determines serum concentrations of clozapine; however, for some of the adverse effects associated with clozapine therapy, increased norclozapine level appeared to be as an important risk factor as exaggerated clozapine concentration. Low CYP3A4 expression evoking low clozapine-metabolizing capacity was demonstrated to frequently lead to increased clozapine concentrations over the upper limit of therapeutic range (> 600 ng/ml) which might have induced elevated fasting glucose levels and might have led to adverse effects, such as constipation or weight gain. Therefore, careful dosing strategy and thorough monitoring of clozapine serum concentration is highly recommended to avoid extremely high levels in patients with low CYP3A4 expression. On the other hand, elevated relative norclozapine concentration associated with normal/high CYP3A4 expression and some additional contribution of normal/high CYP1A2 expression also seemed to predict some metabolic side-effects, such as increased BMI, fasting glucose and triglyceride levels. To reduce the risk of these metabolic side-effects, lowering norclozapine concentration has been suggested by adjunctive fluvoxamine treatment^46^. Although the CYP1A2 and CYP3A4 inhibitor fluvoxamine^50^ can decrease the rate of clozapine metabolism and norclozapine formation, it has been demonstrated to increase steady-state clozapine concentration. Therefore, the fluvoxamine co-medication strategy should include parallel reduction of clozapine dose and careful monitoring of clozapine levels to avoid the marked increase in clozapine concentrations and the risk of worsening of psychosis or side-effects^53^. However, we note that for patients with CYP3A4 low expression, fluvoxamine co-medication may not be considered as a good combination with clozapine therapy, since the intrinsically reduced clozapine-metabolizing capacity can be expected to be blocked by fluvoxamine resulting in exaggerated levels of serum clozapine and increased risk of side-effects.

Several limitations of the present study should be considered. First, some patients were co-medicated with antipsychotics or mood stabilizers that might have influenced CYP expression. Although haloperidol, aripiprazole, risperidone, amisulpride and lamotrigine have not been reported to modify CYP3A4 expression or activity, valproate can transcriptionally induce CYP3A4^54^. Of 96 patients, seven received valproate co-medication; however, none of them was high CYP3A4 expresser, they were all normal expressers. Valproate-induced CYP3A4 expression could nevertheless be measured by CYP3A4 mRNA levels in the patients’ leukocytes informing about hepatic CYP3A4 activity. Second, the patient reported adverse effects were prospectively collected in a sub-population of 65 patients, which might have decreased the statistical power. Third, genetic polymorphisms of 5-HT_2C_, leptin and leptin receptor have been proven to influence weight gain and metabolic syndrome during clozapine treatment^16,55^; however, these pharmacogenetic parameters were not investigated in this study. Since this was the first study that analysed the association between CYP3A4 expression and clozapine-induced side-effects, the statistical analyses were exploratory and no corrections for multiple comparison were applied, which is also a limitation of the present investigation.

### Conclusion

CYP3A4 low expressers appeared to have a higher risk for exaggerated clozapine concentrations, higher glucose levels and BMI, while CYP3A4 normal/high expressers having increased norclozapine/clozapine ratios at the same time showed a risk for elevated glucose and triglyceride levels and abnormal (high) BMI. Additionally, patients expressing both CYP3A4 and CYP1A2 at normal/high levels can be expected to display a higher risk for having high norclozapine/clozapine ratios. CYP3A4 status might therefore be an additional tool for identifying patients with increased risk for these clozapine-induced side-effects. Patients with low CYP3A4 expression may require reduction of clozapine doses, and careful monitoring of metabolic parameters and weight gain is needed during the treatment. Patients with schizophrenia have a higher risk for cardiovascular morbidity and mortality, so it is important to prevent weight gain. For the patients expressing CYP3A4 at normal/high levels and having high norclozapine/clozapine ratios, clozapine metabolism reducing co-medication with careful clozapine dosage or alternative antipsychotic therapy could be considered. Decreasing the risk of metabolic side-effects may also result in better compliance and may lower the cost of treatment.

## Abbreviations

BMI: Body Mass Index
CL: Confidence limits
CYP: Cytochrome P450
GAPDH: Glyceraldehyde 3-phosphate dehydrogenase
HDL: High density lipoprotein
LDL: Low density lipoprotein
OR: Odds ratio
PANSS: Positive and Negative Syndrome Scale
